# Large room-temperature tunneling anisotropic magnetoresistance and electroresistance in single ferromagnet/Nb:SrTiO_3_ Schottky devices

**DOI:** 10.1038/s41598-018-19741-z

**Published:** 2018-01-22

**Authors:** Alexander M. Kamerbeek, Roald Ruiter, Tamalika Banerjee

**Affiliations:** Physics of Nanodevices, Zernike Institute for Advanced Materials, University of Groningen, Nijenborgh 4, 9747 AG Groningen, The Netherlands

## Abstract

There is a large effort in research and development to realize electronic devices capable of storing information in new ways - for instance devices which simultaneously exhibit electro and magnetoresistance. However it remains a challenge to create devices in which both effects coexist. In this work we show that the well-known electroresistance in noble metal-Nb:SrTiO_3_ Schottky junctions can be augmented by a magnetoresistance effect in the same junction. This is realized by replacing the noble metal electrode with ferromagnetic Co. This magnetoresistance manifests as a room temperature tunneling anisotropic magnetoresistance (TAMR). The maximum room temperature TAMR (1.6%) is significantly larger and robuster with bias than observed earlier, not using Nb:SrTiO_3_. In a different set of devices, a thin amorphous AlO_x_ interlayer inserted between Co and Nb:SrTiO_3_, reduces the TAMR by more than 2 orders of magnitude. This points to the importance of intimate contact between the Co and Nb:SrTiO_3_ for the TAMR effect. This is explained by electric field enhanced spin-orbit coupling of the interfacial Co layer in contact with Nb:SrTiO_3_. We propose that the large TAMR likely has its origin in the 3d orbital derived conduction band and large relative permittivity of Nb:SrTiO_3_ and discuss ways to further enhance the TAMR.

## Introduction

Metal-oxide memristive devices provide a promising platform for next generation non-volatile memory and neuromorphic computing architectures^1^. A key feature of a memristive device is its non-volatile variable resistance which depends on the history of the applied current or voltage bias. An important example is the Schottky junction which forms at the interface of noble metals and Nb-doped SrTiO_3_ (Nb:SrTiO_3_), which has shown large and strongly tunable electroresistance (ER)^2–6^. Besides memristive devices, magnetic material based memories such as magnetic random access memory are also strong contenders for next generation data storage devices^7^.

The realization of heterostructures which *simultaneously* exhibit electro- and magnetoresistive effects is of great interest, for instance to realize closer integration of memory and (multi-bit) memory/logic. Although there have been significant advances, the heterostructures used are often composed of complex materials such as multiferroics. These multiferroics need dedicated deposition tools and only operate reliably at low temperatures^8–11^. Additionally such devices use a very thin spacer layers that acts as a tunnel barrier, allowing a tunneling magnetoresistance effect. On top of the mangetoresistance effect also an electroresistive (ER) effect is present in these devices. The electroresistive effect can be switched between a high and low (non-spin selective) resistance state via an electric field. The requirements for these electro- and mangetoresistance effects often compete. For instance the electroresistive switching operates by opening/closing of filamentary conduction paths. Such filaments effectively short the junction and hence removes or reduces the magnetoresistance.

In this paper we demonstrate the simultaneous occurrence of electro- and magnetoresistive effects at room temperature by studying the phenomenon of tunneling anisotropic magnetoresistance (TAMR) in Nb:SrTiO_3_ tunneling devices. In these devices the Schottky barrier acts as the tunnel barrier, removing the necessity of an ultra thin insulating layer. TAMR originates from spin orbit coupling effects. It manifests itself as a change of the junction tunnel conductance when the magnetization is rotated with respect to the current flow direction^12^ or the crystallographic axis^13^. By interfacing the 3*d* transition-metal ferromagnet Co with Nb-doped SrTiO_3_ (a 3*d* conduction band semiconductor), we realize devices which simultaneously exhibit a large TAMR and ER at room temperature. By utilizing the ER, an on/off ratio of over 10^3^ is realized. Additionally, the resistance of the junction can be gradually varied by controlling the amplitude of the voltage cycling. Further, these junctions manifest a TAMR effect of up to 1.6% at room temperature, much larger than reported in other systems (≤0.3%)^14–21^. Uniquely, the TAMR effect is observed to increase with increasing negative bias while in conventional TAMR devices a monotonous decrease is generally observed. We also show that the ER and TAMR effects are strongly suppressed by insertion of an amorphous 1.1 nm thick AlO_x_ layer in between the Co/Nb:SrTiO_3_ interface. This points to the importance of the Schottky barrier and the hybridization of the Co and Nb:SrTiO_3_ orbitals for the observation of both effects.

## Results

### Tunneling anisotropic magnetoresistance

A 20 nm thick Co layer is grown on top of Nb:SrTiO_3_ which is processed into devices with junction areas from 5000 up to 40 000 μm^2^, see for more details the method section. The data presented is from a junction with an area of 20 000 μm^2^. In order to make sure that the main voltage drop is always over the Schottky barrier, we stay well within the range where this is the case. (The lowest Schottky resistance where TAMR is performed is at around 1 MΩμm^2^). The sheet resistance of the semiconductor is on the order of 20 mΩ and the in-plane resistance of the Co contact is well below this. Hence the measurement are all performed in a regime where the interface resistance due to the Schottky barrier dominates the total resistance.

We first investigate the magnetoresistve properties of these Schottky tunnel junctions. At an interface Rashba spin orbit coupling (SOC) is present, since the structure inversion symmetry is broken and a large electric field gradient exist due to the depletion of carriers at the Nb:SrTiO_3_ interface^22^. This Rashba SOC leads to a change of the density of states of the interfacial Co/Nb:SrTiO_3_ layer when the magnetization is rotated with respect to the current flow direction^12^ or the crystallographic axis^13^. This effect is known as tunneling anisotropic magnetoresistance (TAMR).

**Figure 1:**
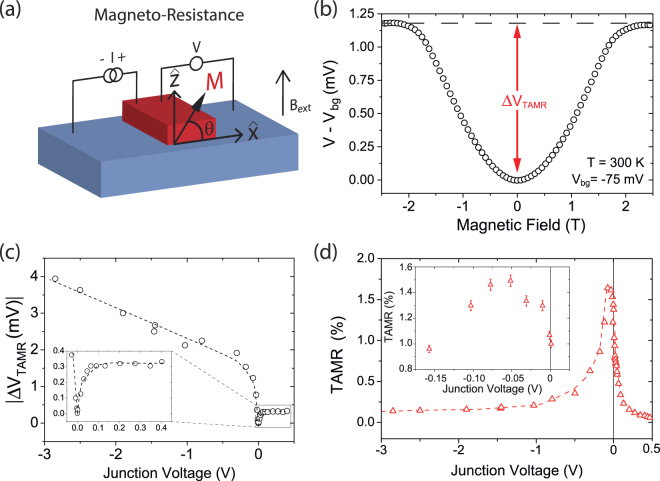
(**a**) The 4-probe TAMR measurement scheme. (**b**) Room temperature TAMR measurement, where a (magnetic field independent) junction voltage (V_bg_ in the figure) of −75 mV has been subtracted. The definition of the TAMR amplitude (Δ*V*_TAMR_) is graphically displayed in red. (**c**) Amplitude of the TAMR effect as function of junction voltage. The inset shows a zoom in of the low bias section (**d**) TAMR amplitude in percentage as function of junction voltage. The TAMR effect does not show a monotonous decay as function of junction voltage as often observed, but peaks at −75 mV to 1.6% as can be seen more clearly in the inset.

The TAMR of the junction is measured by sourcing a constant dc current and measuring the 4-probe voltage as a function of an out-of-plane applied magnetic field B_ext_ (see Fig. 1a). Hence at a certain bias current we report the background junction voltage (defined as *V*(*B* = 0)) as well as the TAMR amplitude, which is the junction voltage change due to the magnetic field. A 4-probe geometry is often employed to measure the TAMR effect^14,18^.

Due to shape anisotropy, the magnetization initially points in-plane (*x*-direction). When B_ext_ is applied the magnetization of the cobalt layer (*M*) rotates out-of-plane with an angle *θ*. Full rotation of the magnetization occurs at a field of around 1.8 T. A typical room temperature magnetic field response of the junctions is shown in Fig. 1b. A parabolic MR is observed which saturates around 1.8 T and a change in junction voltage of 1.2 mV is observed (at a magnetic field independent background voltage of −75 mV). As expected the TAMR has a parabolic dependence on the applied field since the rotation of the magnetization depends quadratically on the magnetic field. Additionally, as expected for TAMR, the effect saturates when the magnetization has fully rotated out-of-plane. Note that anisotropic MR is ruled out, for instance, because the resistance change is much too large (>10 kΩ at low bias) and would not result in the strongly asymmetric bias dependence. The angle dependence of the TAMR at high magnetic field is discussed in Supplementary Fig. S3. Additionally, the absence of in-plane TAMR is shown in Supplementary Fig. S4.

The amplitude of the TAMR effect (Δ*V*_TAMR_), defined as |*V*(*θ* = 0°) − *V*(*θ* = 90°)|, is plotted as function of the junction voltage in Fig. 1c. Note that we plot the TAMR amplitude versus the measured junction *voltage* and not the bias *current*. This was done because the TAMR amplitude scales with the junction voltage (which is proportional to the interfacial electric field). The TAMR amplitude shows a very rapid scaling at low voltage (around ±50 mV) for both polarities, as shown in the inset. However, above +50 mV the amplitude saturates, while at negative bias a linear relation between junction voltage and the TAMR amplitude is observed. This linear scaling continues up to at least −8 V (not shown). In Fig. 1d the TAMR percentage (*V*(*θ* = 0°) − *V*(*θ* = 90°)/*V*(*θ* = 0°) × 100) is shown as a function of junction voltage. It increases for small negative biases while it reduces for positive bias. The TAMR reaches a maximum of 1.6% at −75 mV as can be seen more clearly in the inset, beyond this voltage it starts reducing again. The low bias TAMR(%) as a function of junction voltage is shown for more junctions and with more measurements points in Supplementary Fig. S5b. The reduction is significantly faster in the forward bias direction (+*V*) compared to the reverse bias direction (−*V*). The non-monotonous dependence of the TAMR at reverse bias is of great interest since the TAMR effect is generally seen to rapidly and monotonously decay with increasing bias in either direction^23–28^.

The strong asymmetry of the TAMR in our system is consistent with the, bias dependent, charge transport process across the barrier. When a positive bias is applied the Schottky barrier height is lowered and electrons will not only tunnel through the Schottky barrier, but also transmit over the barrier via thermionic emission. At negative bias the Schottky barrier is not lowered and only tunneling transport can occur. Hence, the TAMR can be expected to decay faster with forward bias. The maximum TAMR is offset from zero bias by −75 mV, which likely relates to the exact details of the tunneling process through the Schottky barrier. The bias dependence of the TAMR is similar for other measured devices including the offset, as shown in Supplementary Fig. S6. Hence by tailoring the electrostatic landscape it might be possible to increase the voltage offset of the TAMR maximum.

### Electroresistance

The same junction that exhibit the TAMR effect as discussed above also exhibits an electroresistive (ER) effect, as is often observed in Nb:SrTiO_3_ Schottky junctions. The observed ER behavior is similar to those reported by others^4^. However by employing ferromagnetic contacts, instead of commonly used noble metal contacts such as Au or Pt, the ER is observed in conjunction with the earlier discussed TAMR.

**Figure 2:**
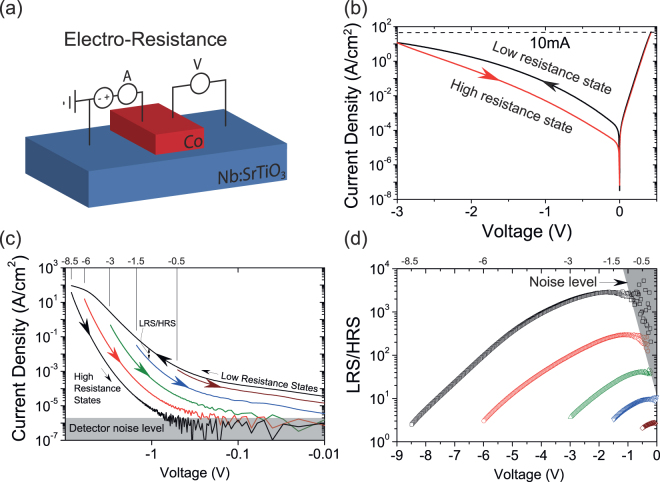
(**a**) Schematic of the 4-probe measurement scheme for the *I*–*V* and electroresistance measurements of the Co/Nb:SrTiO_3_ junction. (**b**) Typical room temperature *I*–*V* measurement showing rectification and large hysteresis when sweeping towards a large negative (reverse) bias (current compliance set at 10 mA). (**c**) A set of five negative bias sweeps. The HRS can be tuned gradually by controlling the maximum negative voltage of the sweep. Between each sweep the device is reset to the low resistance state (LRS) by sweeping to 1 V. (**d**) LRS/HRS ratio versus junction bias. The electroresistance is defined as the ratio of the current of the LRS current (upper branch) divided by the HRS current (lower branch) as shown for the 5 different sweeps in panel (c).

To characterize the electroresistive effects of the devices a 4-probe, 3-terminal measurement geometry is employed as shown in Fig. 2a. This geometry ensures only the contact resistance of the central junction is probed, eliminating the series resistance from the leads and the semiconductor bulk. Figure 2b shows a typical hysteretic *I–V* response of a Co/Nb:SrTiO_3_ junction. The voltage is swept from positive (+0.45 V) to negative (−3 V) voltage (black) and back (red) with a 10 mA current compliance. When sweeping from a positive to negative bias the device is in the low resistance state (LRS, upper branch of the *I*–*V*), and in the high resistance state (HRS) when sweeping back. The charge transport occurs primarily via thermally assisted tunneling. This was determined by the analysis of temperature dependent *I*–*V* measurements in a similar way as done in^29^. From this an *E*_00_ ≈ 13.5 meV is obtained indicating thermally assisted tunneling. Many junctions show similar *I*–*V* characteristics (see Supplementary Fig. S1).

The magnitude of the electroresistance can be precisely controlled by the maximum negative voltage of the sweep as shown in Fig. 2c. The junction is initially in a low resistance state (LRS). When sweeping to a negative voltage and back to zero the device resistance is increased depending on the maximum negative bias of the *I*–*V* cycle. To define the effect size the ratio of the LRS current over the HRS current is plotted in Fig. 2d. In this figure the ratio is displayed for the five different sweeps shown in panel c. A maximum ratio of ~3000 at −1.5 V is found when sweeping to −8.5 V. The large ratios persist down to a low bias of −100 mV, suitable for readout purposes. Note that for the sweeps towards a large negative bias, the HRS junction current at −100 mV is below the detector sensitivity and thus it is not possible to define the LRS/HRS ratio. The junctions show no degradation of the ER effect during a full day of continuous cyclic *I*–*V* measurements to −8.5 V (see Supplementary Fig. S2).

While the main finding in the manuscript is the sizable TAMR effect its presence in combination with the ER effect is of great interest as it open up the possibility of two, simultaneously accessible, state variables in which to store information.

### Interplay between TAMR and Electroresistance

**Figure 3:**
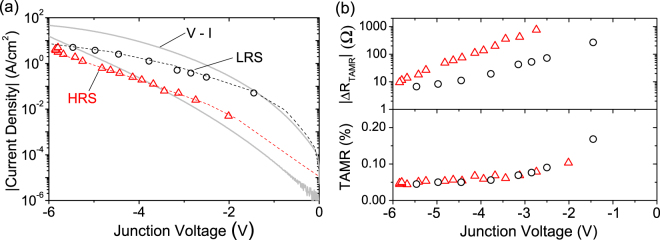
(**a**) Current density versus average background junction voltage during the LRS-TAMR measurements (black circles) and HRS-TAMR measurements (red triangles). For reference an *J*–*V* measurement from Fig. 1c is shown in light gray. (**b**) (top panel) The junction resistance change due to the TAMR effect, (Δ*R*_TAMR_ = Δ*V*_TAMR_/*I*_bias_). The junction voltages of the TAMR measurements correspond with the data which is shown in panel (a). Hence, the corresponding bias current for each HRS/LRS TAMR measurement can be found in panel (a). (bottom panel) TAMR(%) as function of junction voltage. The collapse of all data points on a single line shows that the TAMR effect is *independent* of the ER switching.

To investigate whether there exists a coupling between the electroresistance and the TAMR effect, TAMR measurements were performed in both the low and high resistance states. The low resistance state (LRS) was set by first applying a positive junction current with a compliance limit of 10 mA, to reset the state. Then a certain negative bias current was applied, which results in a certain (magnetic field independent) junction voltage. After setting the negative bias current, a TAMR measurement was performed. In Fig. 3a the black circles denote at which junction voltage and corresponding background current density a LRS-TAMR measurement was recorded. Hence 8 LRS TAMR measurements are collected.

Similarly as for the LRS-TAMR measurements, TAMR measurements were performed in the HRS resulting in a set of 17 TAMR measurements for which the junction was in the HRS. Note that to set the junction in a HRS we directly apply a negative bias current (so no positive bias current reset step), the size of the current is chosen such that it results in a junction voltage of ~−6 *V*. After setting the junction in the HRS, the bias current was reduced to the desired value. The biases at which a HRS-TAMR measurement were performed are plotted in 3a as red triangles. Due to drift in the junction voltage we report the average voltage during a TAMR measurement. Notice that the black circles (red triangles) effectively form the LRS (HRS) branch of the *J*–*V* measurement, as shown in light gray in 3a. The reason that the current density of a LRS-TAMR measurement is lower than it would be for a regular *J*–*V* measurement is due to the aforementioned drift. The drift is caused by the sustained negative bias current during a LRS measurement, which forces the junction towards a HRS.

It is now possible to compare the TAMR effect when the junction is in the HRS and LRS. This is shown in the top panel of Fig. 3b. Here the resistance change due to the TAMR effect (Δ*R*_TAMR_ = Δ*V*_TAMR_/*I*_bias_) is displayed for both the HRS and LRS TAMR measurements as a function of the junction voltage. This shows that the change in junction resistance due to the TAMR effects is different and thus depends on whether the junction is in the HRS or LRS state. This can be understood by realizing that a different current density through the junction is needed to reach a certain junction voltage when the junction is in the HRS or LRS. i.e. a lower current density is needed in the HRS state compared to the LRS to reach the same junction voltage. However, this is a direct consequence of the electroresistive effect and not a change in the TAMR effect due to the electroresistance effect. This is shown in the bottom panel of Fig. 3b, where the same TAMR measurement results are now displayed as a TAMR percentage (this is obtained by dividing the top panel data by the junction resistance (*V*/*I*). Whether the junction is in the HRS or LRS, the TAMR effect in percentage is the same.

The TAMR effect is thus independent of the current density through the junction and thus unaffected by the ER switching. Rather it is controlled by the junction voltage, as it remains the same in both resistance states, as long as the junction has the same bias voltage. This shows that the TAMR amplitude is independent of the physical changes which allow the ER effect. This independence places restrictions on the possible mechanism(s) that are responsible for the ER effect in our devices. Since TAMR relies on tunnel conduction, the ER can not open up an additional pathway in which charge transport, other than tunneling, occurs (e.g. filamentary switching). In general it is unlikely that additional conduction pathways are opened/closed by ER switching since this should at least affect the TAMR amplitude (e.g. opening/closing of resonant tunneling conduction paths).

## Discussion

As the observation of TAMR at room temperature is a major finding in the manuscript we will mainly focus our discussion on the TAMR effect as well as its interplay with the ER switching. The room temperature TAMR size of 1.6% is much larger than observed for low spin-orbit coupling (SOC) strength transition metal ferromagnet based tunnel junctions (≤0.3%)^14–21^. It is also much larger compared to antiferromagnetic (AFM) based TAMR devices using IrMn, where the heavy element Ir has a much larger SOC. Although a TAMR of 160% was observed at low temperature it vanished above 100 K^30^. Room temperature TAMR was achieved by a different group using Pt/Co multilayers in contact with AFM IrMn, but a maximum effect of 0.24% was observed^17^.

Since we employ the common transition metal ferromagnet Co, the enhancement in the TAMR effect likely originates from the use of Nb:SrTiO_3_ as counter electrode. This enhancement can be caused by several mechanisms. Firstly, the TAMR effect requires tunneling transport through the interface for which a narrow enough Schottky barrier is needed. The width of the depletion region *W* is determined by the relative permittivity *ε*_r_ and doping density *N* of a material and is proportional to (*ε*_r_/*N*)^0.5^. Since Nb:SrTiO_3_ has a *ε*_r_ ~ 300 at room temperature, a significantly larger doping density is needed to facilitate tunneling transport compared to for instance n-GaAs with an *ε*_r_ ~ 10. The higher doping results in a larger depletion charge in the depleted Nb:SrTiO_3_ region. At the same time, an opposite but equal charge is present on the Co electrode, which is screened within a few unit cells inside the Co. This results in a large electric field at the surface of the Co layer. The electric field is altered by applying a bias which is expected to affect the TAMR effect by altering the interfacial Rashba SOC strength.

A second cause for the enhanced TAMR could originate from the orbital hybridization of Co and Nb:SrTiO_3_. There is both experimental and theoretical work that indicates the 3*d* orbitals contribute significantly to the tunnel current through a SrTiO_3_ barrier^31–33^. Since the 3*d* states are more confined and have a larger SOC, a larger TAMR is expected compared to systems where the Co *s*-states are more dominant in the conduction (such as for Al_2_O_3_ tunnel barriers). Additionally, it has been shown that the 3*d* orbital nature of the Nb:SrTiO_3_ conduction band can bond with the 3*d* band of Co, such that the conductance of minority spins is much larger than the majority spin of the Co^31^. The tunnel conductance is more sensitive to density of states changes when only one spin type contributes to the tunneling transport.

Finally, it has been theoretically predicted that due to hybridization and charge transfer, a large change in the magneto crystalline anisotropy is expected for the interfacial Co layer on top of SrTiO_3_^34^. Since SOC is at the basis of magneto crystalline anisotropy this suggests that the SOC strength at the interfacial Co layer should indeed strongly differ from the bulk Co. To clarify the (relative) relevance of the previously mentioned mechanisms in-depth investigations into for instance the role of orbital hybridization is needed, both experimentally and theoretically.

**Figure 4:**
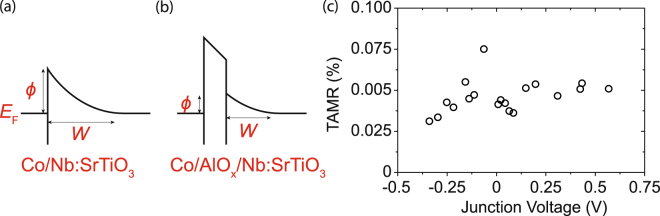
(**a**) Energy diagram of a Metal/Nb:SrTiO_3_ interface and (**b**) the effect of insertion of a thin insulating tunnel barrier. The AlO_x_ tunnel barrier causes a large reduction in the Schottky barrier height (*ϕ*) and the depletion region width (*W*). This reduction is due to the redistribution of the interface potential which now partly drops over the insulator which reduces the electric field at the interface. (**c**) The TAMR amplitude measured for a Co/AlO_x_ (~10 Å)/Nb:SrTiO_3_ device as function of junction voltage.

Although such an in-depth investigation is outside the scope of this work we substantiate the aforementioned propositions by investigating devices with a thin amorphous ~1 nm AlO_x_ layer inserted between the Co and Nb:SrTiO_3_ interface. The AlO_x_ layer separates the Co and Nb:SrTiO_3_ orbitals, thereby suppressing hybridization and charge transfer effects. Additionally, it reduces the height and width of the Schottky barrier and thus the electric field at the surface of the Co layer as schematically shown in Fig. 4a,b. This works particularly well when the relative permittivity *ε*_r_ of the insulating layer is much lower than the semiconductor, as is the case for AlO_x_ (*ε*_r_ ≈ 10) and Nb:SrTiO_3_ (*ε*_r_ ≈ 300)^29^.

Figure 4c shows the extracted maximum TAMR amplitude as function of junction voltage for a Co/AlO_x_(11 Å)/Nb:SrTiO_3_ junction. Compared to the Co/Nb:SrTiO_3_ Schottky junctions the maximum possible TAMR in these junctions is at least 2 orders of magnitude smaller. Additionally, the ER effect in these junctions is orders of magnitude smaller as well (not shown). Note that although the overall junction resistance for these devices is significantly lower than the Co/Nb:SrTiO_3_ Schottky junctions, the charge transport mechanism is also tunneling dominated^29^. We have additionally performed similar measurements with non-magnetic electrodes on Nb:SrTiO_3_ and do not find such a room temperature MR effect.

These Co/AlO_x_/Nb:SrTiO_3_ devices were also studied earlier in our group, to investigate the injection and detection of a spin accumulation into Nb:SrTiO_3_^35,36^. The presence of such a spin accumulation results in an anisotropic Lorentzian MR effect (the Hanle effect) when a magnetic field is applied out-of-plane. It has been shown before that on top of the Hanle effect it is possible to observe TAMR as well. This manifests itself as an anisotropy of the junction MR at very high fields when the magnetic field is applied in-plane versus out-of-plane^37^. Although it is not possible to determine the exact amplitude of the TAMR effect, since other effects such as an anisotropic spin lifetime or anisotropic tunnel spin polarization will result in a similar anisotropy, it is possible to quantify the upper possible limit of the TAMR effect for those junctions (see Supplementary Fig. S6).

Finally, we discuss the interplay between the TAMR effect and ER switching. The TAMR effect is observed both for the LRS or HRS state of the device, as shown in Fig. 3. The electroresistive switching is not observed to alter the TAMR percentage. Besides the TAMR, it could in principle be expected that the magnetic anisotropy of the Co layer is dependent on the electroresistive state of the junction. It has been shown that the magnetic anisotropy can be altered by electric field^38–40^. This is of great interest as electric control over the magnetic state could lead to very fast and energy efficient magnetic switching. Rotation of the magnetic anisotropy from in-plane towards the out-of-plane direction can be experimentally observed in a TAMR measurement. This is because the TAMR lineshape is only quadratic when the applied magnetic field is along the magnetic hard axis and orthogonal to the magnetic easy axis. A change in the magnetic anisotropy in the out-of-plane direction could thus be detected by a change in TAMR lineshape. However, we do not observe any change in the TAMR lineshape between the LRS-TAMR and HRS-TAMR measurements. This indicates that there is no significant change in the magnetic anisotropy of the interfacial Co layer due to electroresitive switching. We do note that the rotation of the magnetization should be relatively large (>15°) to result in changes of the TAMR lineshape that are well noticeable experimentally. This insensitivity could be explained by noting that the electric field only penetrates into the first few atomic layer of the Co at the interface with Nb:SrTiO_3_. Hence it is possible that the bulk magnetization of the 20 nm thick Co layer magnetically pins the magnetization of the interfacial Co layer. If this is the case, reduction of the magnetic layer down to only a few atomic layers might realize a coupling between the magnetic and electric states.

## Conclusion

In conclusion, we find a sizable TAMR at room temperature, in combination with ER effects for Nb:SrTiO_3_ Schottky junctions with ferromagnetic Co contacts. The TAMR effect is as large as 1.6% in these junctions at room temperature. Additionally, we show the simultaneous occurrence of electroresistive switching along side the TAMR. While the ER effect by itself has been reported before in Nb:SrTiO_3_ Schottky junctions its simultaneous existence with the TAMR effect was not. The combined occurrence is of great value since it is very challenging to create devices that exhibit both MR and ER simultaneously. The ER effect allows for a gradual variation of the device resistance by over 10^3^. The room temperature TAMR effect size is much larger than those reported in literature and has an unconventional bias dependence. The origin of this enhancement likely originates from the large permittivity of Nb:SrTiO_3_ and hybridization of the Co and Ti *d*-orbitals. The hypothesis of the importance of the built-in electric field at the Co interface layer and intimate contact of Co with Nb:SrTiO_3_ has been strengthened by insertion of an amorphous ~1 nm AlO_x_ layer which significantly reduced both ER and TAMR effects. These effects have been realized in devices which do not require precise stoichiometry of ultra-thin ferroelectric or multiferroic layers. Further enhancement of the ER and TAMR effect size is expected by: (1) optimizing the electrostatic landscape of the devices, (2) down scaling to smaller dimensions, (3) using heavy metal based (anti-)ferromagnets and (4) a better understanding of the role of orbital effects. The simultaneous occurrence of ER and TAMR shows considerable prospects for complex oxide based devices which combines both spin and charge as state variables.

## Methods

The ferromagnetic Schottky junctions were grown by electron-beam evaporation of Co (20 nm) at a base pressure of ~10^−6^ Torr and at room temperature. Prior to deposition, the Nb:SrTiO_3_ crystals (0.1 wt% Nb) were TiO_2_ terminated via a wet etching protocol^41^ (but not annealed), followed by *in*-*situ* oxygen plasma cleaning. Junctions were fabricated using UV-photolithography and ion beam etching and have sizes ranging from 5000 to 40000 *μ*m^2^.

## Electronic supplementary material

Supplemental Material: Large room-temperature tunneling anisotropic magnetoresistance and electroresistance in single ferromagnet/Nb:SrTiO$_3$ Schottky devices.
