# Supplementary material for: Large room-temperature tunneling anisotropic magnetoresistance and electroresistance in single ferromagnet/Nb:SrTiO3 Schottky devices
Source: Sci Rep. 2018 Jan 22;8:1378. doi: 10.1038/s41598-018-19741-z (PMC5777995; doi:10.1038/s41598-018-19741-z)
Supplement: Supplementary file 1 — Supplemental Material: Large room-temperature tunneling anisotropic magnetoresistance and electroresistance in single ferromagnet/Nb:SrTiO$_3$ Schottky devices. [file 41598_2018_19741_MOESM1_ESM.pdf]

Supplemental Material: Large room-temperature tunneling anisotropic magnetoresistance and electroresistance in single ferromagnet/Nb:$SrTiO_3$ Schottky devices.

A. M. Kamerbeek,[1] R. Ruiter,[1] and T. Banerjee[1,*]

[1] *Physics of Nanodevices, Zernike Institute for Advanced Materials, University of Groningen, Nijenborgh 4, 9747 AG Groningen, The Netherlands*

(Dated: January 1, 2018)

1. JUNCTION TO JUNCTION HOMOGENEITY OF CHARGE TRANSPORT

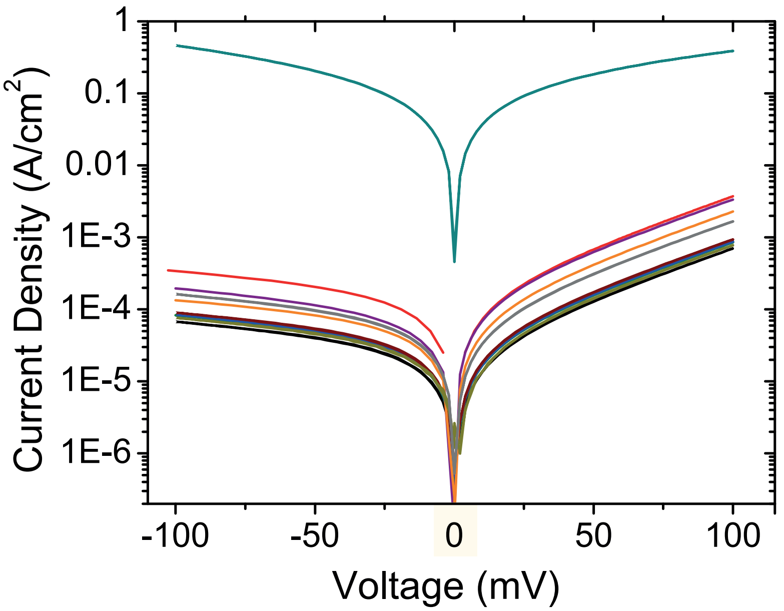

Figure S 1. Junction to junction *I*-*V* homogeneity, junction area spans from 5000 up to 40000 μm^2. The *I*-*V*s of the junctions show both a very similar absolute current density as well as the current density dependence on voltage bias (similar quantitative rectification). A small number of junctions strongly deviate from the observed behavior as shown here for one junction. In this case the junction resistance is always many orders of magnitude lower and does not show rectification.

2. ELECTRORESISTIVE CYCLING ENDURANCE

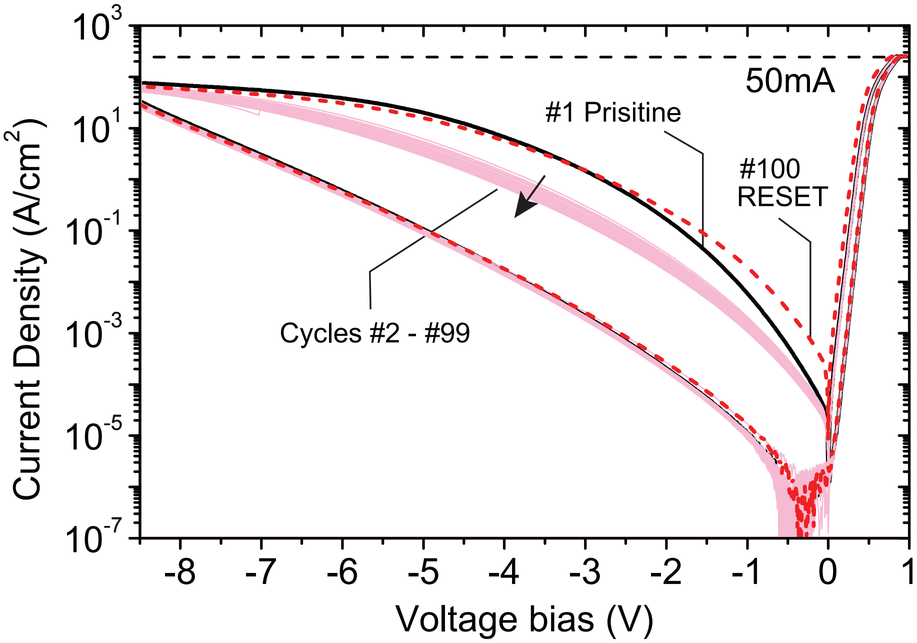

Figure S 2. Electroresistive device durability. 100 cycles up to -8.5 V bias (low bias ON/OFF ratio $\sim 500 - 1000$) showing no degradation of the junction. The solid black line is the first cycle, the dashed red line is cycle nr. 100. Prior to performing cycle nr. 100 the junction was reset to a LRS by a applying a bias of 1 V for 1 hour at 50 mA current compliance. A similar behavior as seen at cycle nr. 1 is obtained. The reduced ER after the first cycle is due to not fully resetting the junction to the LRS as the cyclic I-V only waits 120 seconds at the maximum positive and negative voltages between each cycle. The total measurement time for the displayed data is around 24 hours, each half cycle takes about 5 minutes.

3. ANGLE DEPENDENCE OF TAMR IN CONSTANT 6 T OUT-OF-PLANE MAGNETIC FIELD

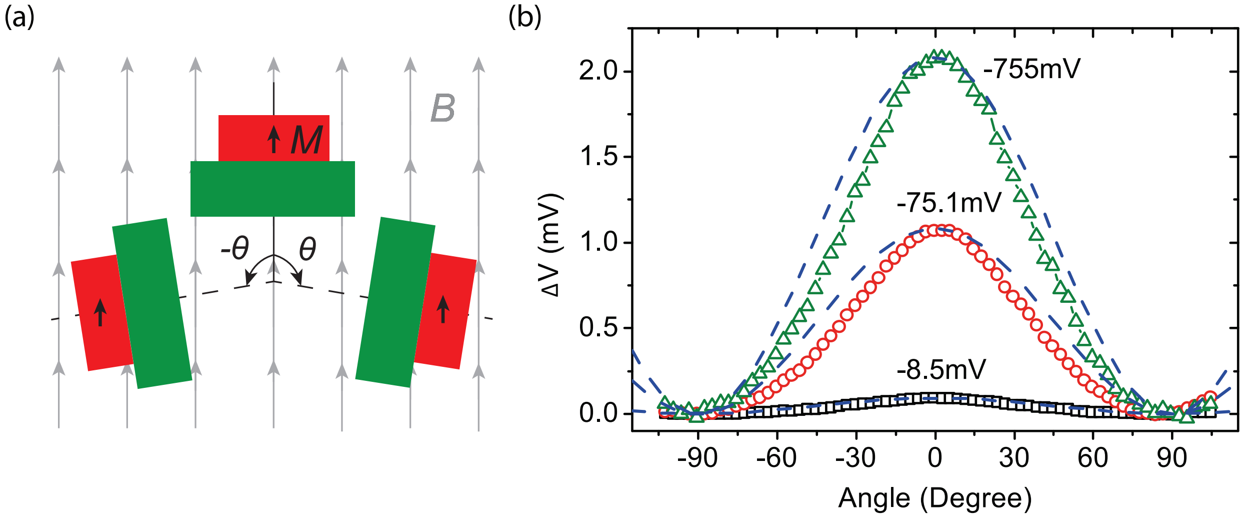

Figure S 3. (a) Experimental setup to measure the angular dependence of the TAMR effect. A large magnetic field B_Z of 6 T is applied perpendicular to the surface normal. The sample is rotated in this field up to an angle θ of $\pm 100°$ (b) Junction voltage as a function of sample angle θ with the applied magnetic field at different junction voltage biases: -8.5 mV (black squares), -75.1 mV (red circles) and -755 mV (green triangles). A magnetic field independent background voltage has been subtracted from the data. A two fold symmetry is observed consistent with the TAMR effect, the blue dashed lines are fits using the expected angular dependence ($V_{TAMR} \times (\cos(2\theta) + 1)$, where the angle θ is with respect to the out of plane direction) of the out-of-plane TAMR effect. A slight deviation between the fit and the data at intermediate angles is observed. This could originate from the fact that the magnetization is not perfectly collinear with the applied field due to the large demagnetization field of the Co layer (around 1.8 T).

4. TAMR WITH AN IN-PLANE VERSUS OUT-OF-PLANE MAGNETIC FIELD

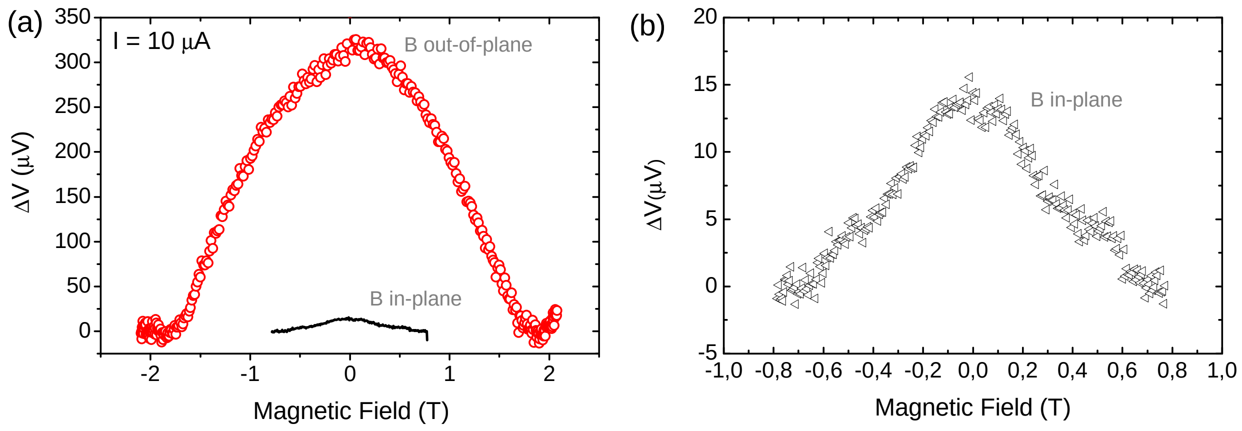

Figure S 4. (a) Averaged TAMR measurement at 10 μA for an in-plane and out-of-plane magnetic field. (b) The same in-plane measurement as in panel (a), but now on a different scale. There is almost no B dependence for the in-plane measurement. The small dependence is caused by the fact that the magnetic field is not perfectly in-plane, thus there is a small out-of-plane B component. The estimated misalignment is $\tan^{-1}\left(\frac{15}{315}\right) \approx 3°$.

5. DEVICE TO DEVICE BEHAVIOR OF ER AND TAMR

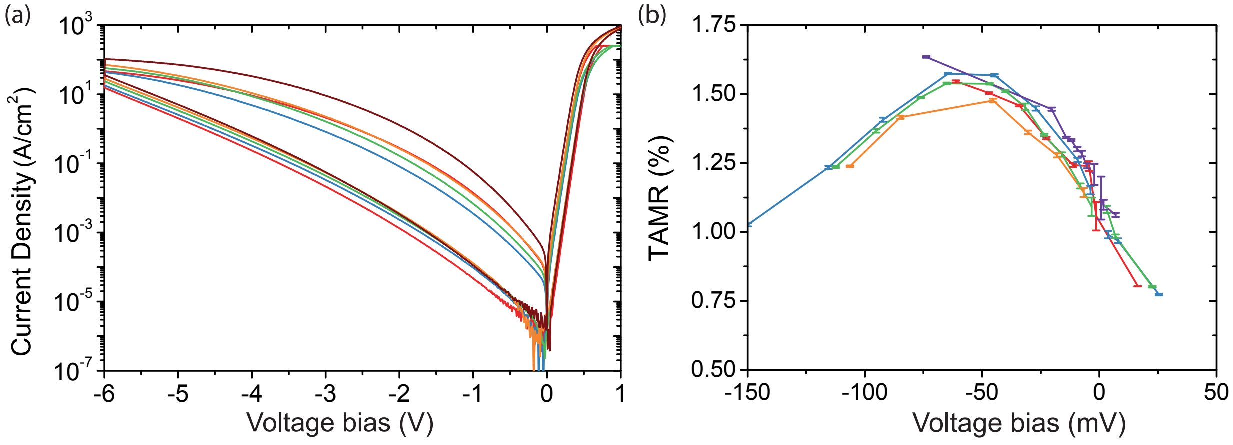

Figure S 5. Bias dependence of (a) electroresistance and (b) TAMR effect for 5 different junctions at room temperature showing very similar ER/TAMR amplitude and bias dependence. The TAMR measurements were all made on pristine devices, meaning no ER cycle had been performed yet. After ER cycling the behavior is the same.

6. DETERMINATION OF MAXIMUM TAMR

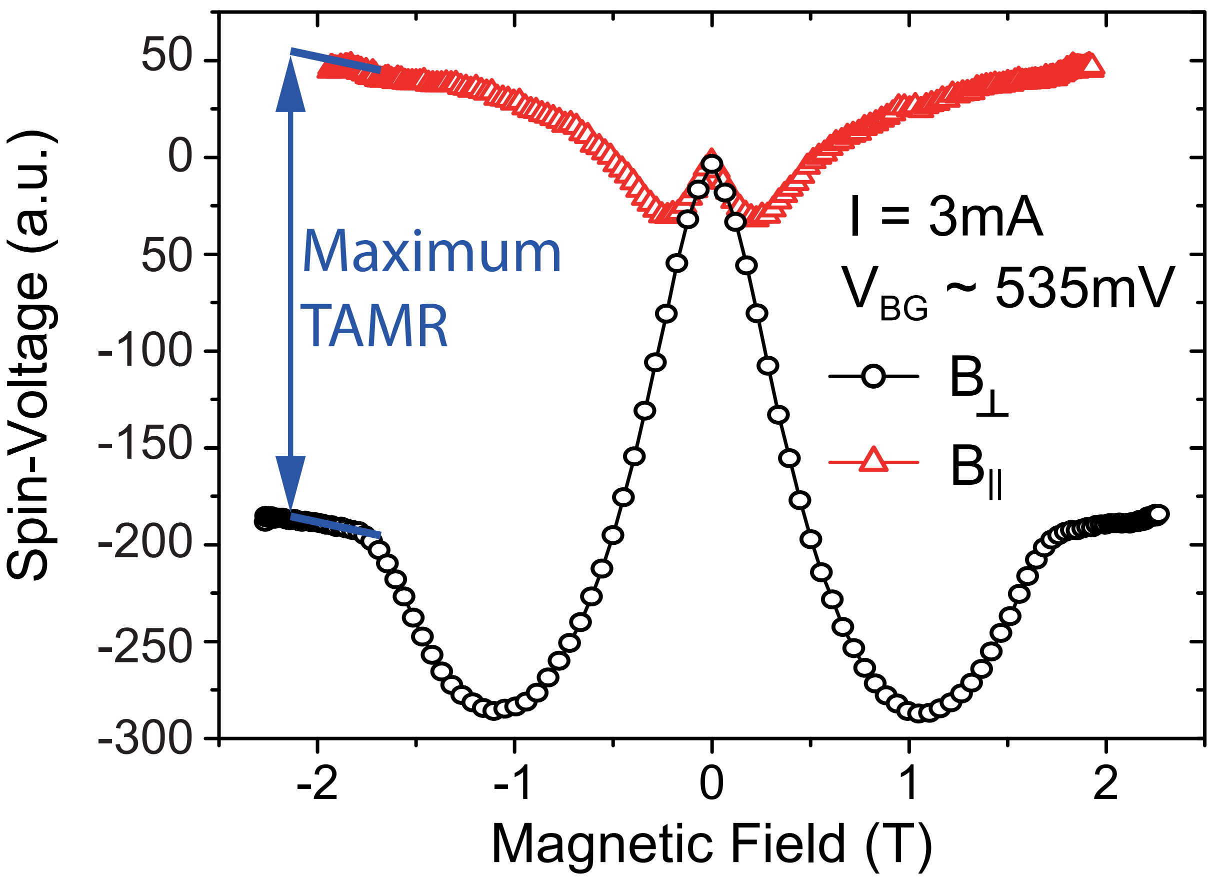

Figure S 6. Magnetoresistance of a Co(20 nm)/AlO_x(∼1 nm)/Nb:$SrTiO_3$ junction at room temperature for out-of-plane applied field (black circles) and in-plane applied field (red triangles). The maximum TAMR can be defined as the voltage difference at large magnetic fields between an in and out-of-plane applied magnetic field. The TAMR is likely smaller since an anisotropic spin lifetime and anisotropic tunnel spin polarization can result in a similar anisotropy. We would like to note that at high magnetic field an additional, isotropic, linear MR is observed.

* t.banerjee@rug.nl
